# A novel de novo MYH9 mutation in MYH9-related disease: A case report and review of literature

**DOI:** 10.1097/MD.0000000000018887

**Published:** 2020-01-24

**Authors:** Qi Ai, Linsheng Zhao, Jing Yin, Lihua Jiang, Qiuying Jin, Xiaoli Hu, Sen Chen

**Affiliations:** a Department of Hematology and Oncology; b Department of Pathology; c Department of Immunology, Tianjin Children's Hospital, Tianjin, China.

**Keywords:** de novo mutation, Döhle-like inclusions, macrothrombocytopenia, MYH9-related disease, NMMHCIIA

## Abstract

**Introduction:**

MYH9-related disease (MYH9-RD) is a rare autosomal dominant disorder caused by mutations in MYH9, which is responsible for encoding nonmuscle myosin heavy chains IIA (NMMHCIIA). MYH9-RD is clinically characterized by congenital macrothrombocytopenia, granulocyte inclusions variably associated with the risk of developing progressive sensorineural deafness, cataracts and nephropathy.

**Patient concerns:**

A 5-year-old boy had a history of a mild bleeding tendency and chronic thrombocytopenia, first identified at four months of age. No other family members were noted to have similar clinical features or hematologic disorders.

**Diagnoses:**

The boy was diagnosed with MYH9-RD. Light microscopic examination of peripheral blood films (Wright-Giemsa stain) showed marked platelet macrocytosis with giant platelets and basophilic Döhle-like inclusions in 83% of the neutrophils. Immunofluorescence analysis disclosed a type II pattern, manifested by neutrophils which contained several circle-to-oval shaped cytoplasmic NMMMHCA-positive granules. Sequencing analysis of MYH9-RD genes was carried out and revealed a novel missense mutation of c.97T>G (p.W33G) in the patient but not in his parents.

**Intervention:**

No treatment is necessary. Recognition of MYH9-RD is important to Avoiding unnecessary and potentially harmful treatments.

**Outcomes:**

The patient's condition remained stable during the follow-up.

**Conclusions:**

As a result of identifying this missense mutation in this particular case, we have added c.97T>G (p.W33G) to the broad spectrum of potential MYH9 mutations.

## 1 Introduction

MYH9-related disorders were initially described as four syndromes, May–Hegglin anomaly, Fechtner syndrome, Sebastian syndrome, and Epstein syndrome. The four conditions are now considered to be heterozygous pathologic variants of the same genetic disorder called MYH9-related disease (MYH9-RD).^[1]^ MYH9-RD is a very rare genetic disease; however, it is one of the most frequent autosomal-dominant forms of macrothrombocytopenia induced by mutations of the *MYH9* gene for NMMHCIIA.^[2]^ Each NMMHCIIA comprises three domains: the head (or motor), neck, and tail domains.^[3]^ Abnormal NMMHCIIA may disrupt the composition and reorganization of cytoskeletons, which may lead to abnormal platelet formation from megakaryocytes, resulting in macrothrombocytopenia.^[4]^ The characteristic clinical features include thrombocytopenia with giant platelets and polymorphonuclear Döhle-like bodies. The patients with MYH9-RD may also display nonhematologic manifestations, including sensorineural deafness, nephropathy, and cataracts.^[5]^

The human *MYH9* gene contains 41 exons spanning ∼33,320 bases and is located on chromosome 22 q12-13.^[6]^ According to a recent review, almost 80 mutations, mostly point mutations, have been reported in the MYH9 pedigrees.^[7]^ The current data demonstrate that there is an obvious genotype–phenotype correlation. Mutations in the motor domain may confer a high risk of bleeding, progressive nephropathy, and deafness.^[8]^

In our case, the diagnosis of MYH9-RD was established by immunofluorescence analysis of a peripheral blood smear. We then identified a novel missense mutation, c.97T>G (p.W33G), through sequencing analysis. The fact that the mutation was not seen in the asymptomatic parents suggested that the mutation was de novo. To the best of our knowledge, this is the first report of a de novo missense mutation, found in the MYH9 of a child with MYH9-RD. The W33G residue is located at the motor domain, which may cause altered extra-hematological manifestations. As expected, our patient had a pathological phenotype compatible with MYH9-RD: macrothrombocytopenia and nephroma with onset at 5 years of age. Based on these findings, the patient requires lifelong follow-up of his hematological and extra-hematological abnormalities.

## 2 Case presentation

In August 2017, a 5-year-old boy was brought to our department for evaluation of persistent thrombocytopenia. The patient had a history of a mild bleeding tendency and chronic thrombocytopenia, first identified at 4 months of age. No other family members were noted to have similar clinical features or hematologic disorders. He had been previously hospitalized three times for fever and thrombocytopenia from July 2015 to February 2017. He was initially diagnosed with idiopathic thrombocytopenia purpura (ITP), thought to be due to an underlying immunologic disorder. However, previous treatment with IVIG and a corticosteroid had failed.

**Figure 1:**
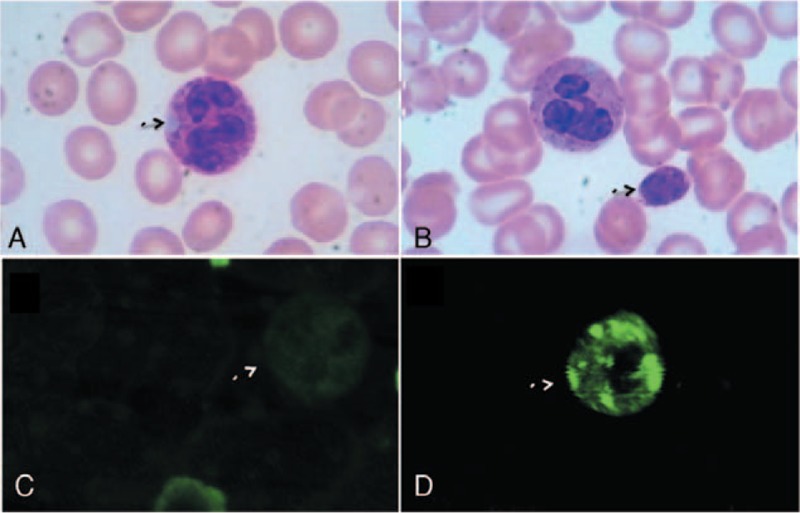
Platelet and neutrophil morphology (original magnification ×1000). (A) Light micrograph of Wright–Giemsa-stained peripheral blood. Typical large inclusion body in a neutrophil is indicated by an arrowhead. (B) Light micrograph of Wright–Giemsa-stained peripheral blood. A giant platelet as large as erythrocyte and a cytoplasmic inclusion body in a neutrophil were observed. (C) Immunofluorescence localization of myosin-9 in neutrophil granulocyte. Normal myosin-9 distribution pattern is shown for comparison. (D) An immunofluorescence micrograph of a neutrophil immunostained with anti-NMMHCIIA localization.

Light microscopic examination of peripheral blood films (Wright–Giemsa stain) showed marked platelet macrocytosis with giant platelets and basophilic Döhle-like inclusions in 83% of the neutrophils (Fig. 1A and B). Because the presence of giant platelets may lead to underestimation of the enumeration of platelets by an automated cell counter, the number of platelets was counted by two different methods (manual and automated counter) simultaneously. The platelet count with the manual method was 41 × 10^9^/L, while the count with an automated counter was 5 × 10^9^/L. There were no significant changes noted in the blood chemistry. The urinalysis showed a slightly positive occult blood but no red blood cells on microscopic evaluation. Ultrasonography demonstrated bilateral, diffusely enlarged kidneys. The audiometric and ophthalmological findings were normal.

We performed immunofluorescence analysis to make a definitive diagnosis of MYH9-RD. The experiments were performed on peripheral blood films with indirect immunofluorescence labelling using the NMMHCIIA rabbit monoclonal antibody (Abcam, Inc, Cambridge, MA). This analysis disclosed a type II pattern, manifested by neutrophils which contained several circle-to-oval shaped cytoplasmic NMMMHCA-positive granules (Fig. 1D). Normal myosin-9 in neutrophil granulocyte distribution pattern is shown for comparison (Fig. 1C).

**Figure 2:**
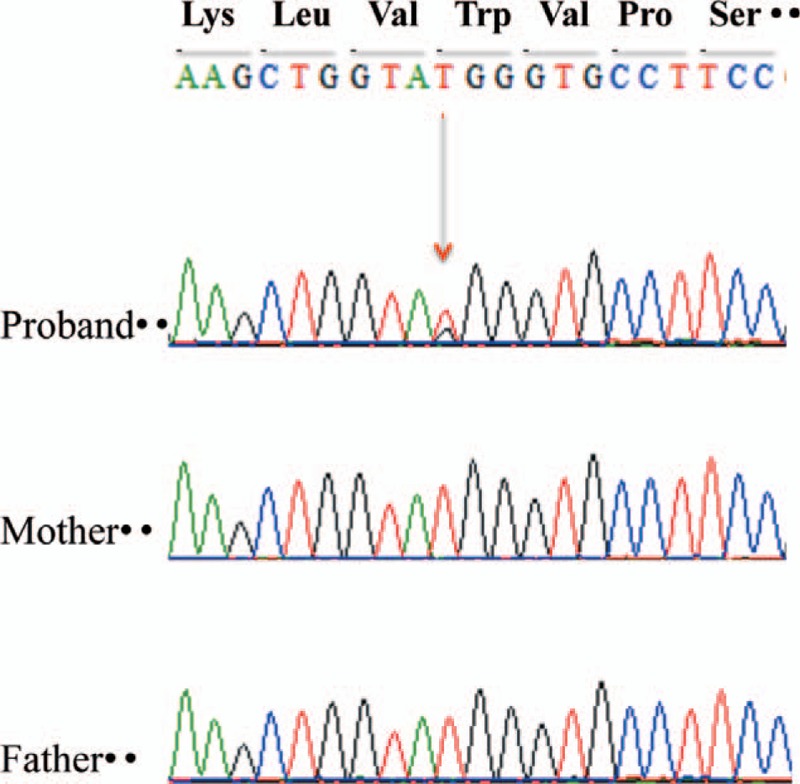
The results of direct sequencing of MYH9 exon 2 in the proband and family numbers. The proband was heterozygous for a missense mutation of c.97T>G (p.W33G). His parents did not have the mutation, indicating a de novo occurrence of the mutation in the proband.

To make a precise diagnosis, we then performed targeted NGS (Next Generation Sequencing) of related *MYH9-RD* genes. Written informed consent of the parents of this child was obtained prior to the collection of their peripheral blood for the following experiment. The study proposal was reviewed and approved by the ethics committee of our hospital. Sequencing analysis of *MYH9-RD* genes was carried out and revealed a novel missense mutation of c.97T>G (p.W33G) in the patient but not in his parents. Since a *MYH9* gene mutation was not identified in either one of his parents, his mutation was thought to be a de novo mutation (Fig. 2). The c.97T>G mutation was not found in dbSNP Allele Freq or ClinVar significant database.

No treatment was given and there were no bleeding complication. This patient was followed up every 3-month. No progression occurred during the 2 years of follow-up.

## 3 Discussion

MYH9-RD is a clinical entity frequently misdiagnosed as ITP because automated blood cell analyzers may miss giant platelets and underestimate the platelet count.^[9]^ Although the presence of granulocyte inclusion bodies on Wright–Giemsa stained peripheral blood films is the hallmark of MYH9-RD, difficulty in detecting the inclusion bodies sometimes leads to misdiagnosis of this disorder. Our case is an example of a misdiagnosis based solely on clinical features and cell morphology. Hence, we believe immunofluorescence analysis or genetic analysis is essential studies for making a correct diagnosis of this disorder.

An immunofluorescence analysis is the most widely used technique for detection of trace amounts of an abnormal NMMHCIIA location. The diagnosis of MYH9-RD can be established in a patient with typical MYH9 protein aggregates in neutrophils detected through immunofluorescence analysis of a peripheral blood film. Conversely, the absence of inclusions excludes the diagnosis of MYH9-RD.^[10]^ Abnormal NMMHCIIA localization was observed in every neutrophil from our patient. In contrast, immunofluorescence analysis of NMMHCIIA in leukocytes of his parents showed normal, diffuse, and homogeneous distribution throughout the cytoplasm. As a result of these findings, we suspected that this case of Fechtner syndrome due to a MYH9 mutation was de novo.

Using sequence analysis, we identified a de novo mutation in MYH9. De novo mutations are not rare events. The average human germline single nucleotide variants (SNVs) mutation rate is estimated to be 1.18 × 10^−8^ per nucleotide or 74 novel SNVs per genome per generation.^[11]^ Previous reports have shown that the percentages of de novo mutations in MYH9 ranged from 20% to 35%.^[12]^

To the best of our knowledge, over 80 different MYH9 mutations have been identified. In the present study, we identified a novel missense mutation and added c.97T>G to the spectrum of MYH9 mutations. The mutation was predicted to be damaging by both SIFT and Polygen-2 software. MYH9 is a large gene with 41 exons, and there are six hot spots of mutation at residues 96 (exon 2), 702 (exon 17), 1165 (exon 27), 1424 (exon 31), 1841 (exon 39), and 1933 (exon 41).^[13]^ The coding region of exons 2 to 20 translates for the head and neck domain of NMMHCIIA and exons 21 to 41 for the tail portion of the protein. The mutation has been identified to be a T-to-G substitution in exon 2, replacing tryptophan of the codon 33 with glycine on the SH3/MD interface region of NMMHCIIA. Further molecular analysis has suggested that tryptophan 33 is an important residue for stabilizing the hydrophobic interface between the motor and SH3-like domains of NMMHCIIA. Previous genotype-phenotype correlation studies showed a higher severity for MD mutations. Patients with SH3/MD interface were noted to present an intermediate-high risk of developing deafness. It was proposed that the SH3/MD substitutions are associated with a high risk of developing sensorineural deafness, whereas the risk of developing cataracts and nephropathy is low.^[14]^ So far, the mutations of the W33 residue include p.W33R and p.W33C. Patients with the p.W33R mutation have macrothrombocytopenia without nonhematologic manifestation,^[15]^ whereas patients with the p.W33C mutation have macrothrombocytopenia and hearing loss.^[16]^ The patient in the present case report showed macrothrombocytopenia and renal impairment. We believe that different mutations at the same residue can have a different impact on the function of this protein. However, further investigation is needed for clarification and confirmation of the genotype–phenotype correlation of these mutations.

## Acknowledgments

We thank Dr Phillip R. Bryant for a helpful critical reading of the article and providing constructive comments.

## Author contributions

**Conceptualization:** Sen Chen.

**Formal analysis:** Jing Yin, Qiuying Jin.

**Investigation:** Xiaoli Hu.

**Methodology:** Jing Yin.

**Project administration:** Qi Ai.

**Resources:** Linsheng Zhao.

**Supervision:** Lihua Jiang.

**Validation:** Sen Chen.

**Visualization:** Sen Chen.

**Writing – original draft:** Qi Ai.

**Writing – review & editing:** Sen Chen.

Qi Ai: 0000-0002-6501-3051.
